# Exercise Intensity and Brain Plasticity: What’s the Difference of Brain Structural and Functional Plasticity Characteristics Between Elite Aerobic and Anaerobic Athletes?

**DOI:** 10.3389/fnhum.2022.757522

**Published:** 2022-02-22

**Authors:** Keying Zhang, Yih-Kuen Jan, Yu Liu, Tao Zhao, Lingtao Zhang, Ruidong Liu, Jianxiu Liu, Chunmei Cao

**Affiliations:** ^1^Division of Sports Science and Physical Education, Tsinghua University, Beijing, China; ^2^Department of Kinesiology and Community Health, University of Illinois Urbana-Champaign, Champaign, IL, United States; ^3^Department of Psychology, Guizhou Minzu University, Guiyang, China

**Keywords:** exercise intensity, aerobic exercise, anaerobic exercise, RS-fMRI, brain plasticity

## Abstract

This study investigated the differences in morphometry and functional plasticity characteristics of the brain after long-term training of different intensities. Results showed that an aerobic group demonstrated higher gray matter volume in the cerebellum and temporal lobe, while an anaerobic group demonstrated higher gray matter volume in the region of basal ganglia. In addition, the aerobic group also showed significantly higher fractional amplitude of low-frequency fluctuation (fALFF) and degree centrality (DC) in the motor area of the frontal lobe and parietal lobe, and the frontal gyrus, respectively. At the same time, the anaerobic group demonstrated higher fALFF and DC in the cerebellum posterior lobe (family-wise error corrected, *p* < 0.01). These findings may further prove that different brain activation modes respond to different intensities of physical activity and may help to reveal the neural mechanisms that can classify athletes from different intensity sports.

## Introduction

Exercise intensity is one of the most concerning components of exercise. Exercise can be divided into aerobic exercise and anaerobic exercise according to the different exercise intensities. Generally speaking, there are three energy systems serving different needs of muscles when individuals are performing motors of different intensities. They are the aerobic energy system, glycolysis energy system, and adenosine triphosphoric acid and phosphocreatine (ATP-CP) energy system, the last two of which are also called the anaerobic energy system (Gastin, 2001). Endurance and sprint performance, which support totally different physical activities, were largely decided by the working capacity of the aerobic energy system and anaerobic energy system, respectively. Specific endurance and strength training can improve the corresponding capacity. Studies have shown that anaerobic and anaerobic training could lead to adaptions in the cardiovascular system (such as cardiac output and stroke volume), muscular system (such as muscle fiber size mitochondrial density), and peripheral nervous system (such as motor units recruitment) (Garcia-Pallares and Izquierdo, 2011; Piacentini et al., 2013). However, only a few preliminary research explored the correction between exercise intensity and the activity of the central nervous system. For example, Bruemmer et al. (2011) tested the exercise-induced effects on brain cortical activity using electroencephalography (EEG) and found that cortical activation patterns depend on exercise mode and intensity. It is worth noting that using EEG to detect brain characteristics has an inevitable shortcoming as EEG has a limited observation of those structures located deep down in the cortical surface.

Fortunately, this problem could be well solved by functional magnetic resonance imaging (fMRI) techniques. fMRI has high spatial resolution and has been widely used in cognitive neuroscience, psychology, and sports science. In order to explore exercise-induced brain plasticity, researchers have done many experiments with elite athletes as subjects (Babiloni et al., 2010; Pezzulo et al., 2010; Chang et al., 2011; Bishop et al., 2013; Callan and Naito, 2014; Kim et al., 2014; Naito and Hirose, 2014). Most of these studies adopt the expert-novice paradigm, aiming to compare the differences in brain plasticity between athletes and non-athletes or novices. The research of the expert-novice paradigm is devoted to speculating on the rules and mechanisms of long-term participation in certain sports [e.g., badminton (Milton et al., 2007; Di et al., 2012), basketball (Park et al., 2006), and diving (Wei et al., 2011)] training affecting brain plasticity. There are few studies aiming at studying the differences in brain plasticity of different sports.

Recent studies have proved that different intensities of 30-min acute exercise have different effects on resting-state functional connectivity (Schmitt et al., 2019, 2020). Resting-state is not a complete and true “rest” but similar to “intrinsic brain activity” or “spontaneous brain activity.” It is used to distinguish from the task-state, for example, moving fingers, doing cognitive tasks, etc. However, it is still unclear whether long-term exercises of different intensities have different effects on the brain morphometry and resting-state functional plasticity. Wenzel et al. tested the speed-specific regions in the brain during fast and slow repeatedly dorsi- and plantarflexions using task-fMRI. The results showed that rapid foot movement was accompanied by increased activation in the cerebellar anterior lobe. Wenzel et al. then compared the gray matter volume of the anterior cerebellar lobe of power athletes and endurance athletes by using the anterior cerebellar lobe as a mask. They found a difference (an uncorrected voxel-level threshold with *p* ≤ 0.001) in gray matter volume in the cerebellar anterior lobe between two groups and have concluded that speed-specific neuro-functional and -structural differences exist between power and endurance athletes in the central nervous system (Wenzel et al., 2014). However, they did not compare the gray matter volume in the whole brain but performed statistical tests within certain brain areas (mask). Schlaffke et al. (2014) claimed that high-intensity endurance exercise seems to be related to changes in the plasticity of the medial temporal lobe. They scanned 13 martial artists, 13 endurance athletes, and 13 non-athletes and found that the endurance group showed higher gray matter volume (GMV) in the hippocampus than the martial arts group. However, they also used a loose threshold (voxel threshold *p* < 0.001, uncorrected) in predefined brain areas (mask) with strong prior hypotheses. In summary, more research is necessary to compare aerobic athletes and anaerobic athletes with stricter thresholds across the whole brain.

This study systematically compared the whole-brain structural and functional plasticity of aerobic athletes and anaerobic athletes for the first time. Based on previous studies, it can be hypothesized that: (1) Brain structural and functional plasticity is different between elite aerobic athletes (who participated in long-term aerobic training) and anaerobic athletes (who participated in long-term anaerobic training), now that it is known that different intensities of acute exercise have different effects on resting-state network (Schmitt et al., 2019, 2020), it can be speculated that long-term and repetitive stimulation can deepen this difference. (2) These differences may lie primarily at the frontal lobe, temporal lobe, and cerebellum. So far, only one study performed a statistical test within the predefined brain areas. Therefore, the research results are limited and different across the studies. This current study conducted analysis within the whole brain, which may lead to the distribution of results in different brain regions.

In order to prove these hypotheses, we recruited two groups of athletes: elite endurance and sprint athletes, who have participated in years of endurance (aerobic) and sprinting (anaerobic) training. Brain images were acquired using MRI with all subjects at resting-state, which could reflect the cumulative effect of long-time experience due to the absence of any special motor or cognitive tasks (Lewisa et al., 2009). We then analyzed the brain gray matter volume, the amplitude of low-frequency fluctuation (ALFF), and degree centrality (DC). Voxel-based morphometry (VBM) analysis is a frequently-used method that can detect the density and volume of brain tissue quantitatively at the voxel level. It can also reflect the differences in brain tissue composition and characteristics. ALFF and DC were used to evaluate the functional plasticity of athletes in this article. ALFF refers to the total power of fluctuation in the low-frequency range (Margulies et al., 2010) and it is used to evaluate the resting-state spontaneous fluctuations in fMRI signal. fractional ALFF (fALFF) was proposed to reduce the sensitivity of ALFF to physiological noise. The specific approach is to remove the power spectrum of the low frequency (0.01–0.08 Hz) segment and divide it by the entire power spectrum (Zou et al., 2008). Although the actual significance of ALFF and fALFF still remain unknown, they are considered to be a promising method for detecting regional signals change of brain spontaneous activity (Zou et al., 2008). DC is a data-driven, graph-theoretical method. DC abstracts the brain into a complex network model and can reflect the characteristics of brain network connection and topological properties (Bullmore and Sporns, 2009). Voxels with a higher DC trend perform better and stronger connections with other regions. ALFF focuses on the spontaneous activity of a certain brain area, while DC emphasizes the connection between a certain brain area and the whole brain. Therefore, the comprehensive use of ALFF and DC can comprehensively investigate the brain functional plasticity characteristics of subjects. These methods have been widely used in brain science studies of athletes (Di et al., 2012; Huang et al., 2017; Zhang et al., 2021).

## Materials and Methods

### Subjects

The current study recruited 50 elite track-and-field athletes. They had engaged in at least 2 years of track-and-field training (6.6 ± 4.0 years, range from 2 to 17 years) and have been certified as national level athletes. None of these athletes had a history of neurological disorders or movement disorders. All subjects completed the scanning. But two of them were eliminated because of too much head motion, leaving a sample of 48 athletes. A group of 23 runners who had participated in aerobic exercise for many years (10,000 meters running, half marathon, athletics 20 km walk) were allocated to the aerobic group (14F/9M, 21.1 ± 2.4 years old, training for 7.9 ± 2.5 years). Meanwhile, 25 sprinters who participated in anaerobic exercise for many years (100, 200, 400, and 110 m hurdle, long jump, triple jump, and javelin) were allocated to the anaerobic group (17F/8M, 21.0 ± 1.7 years old, training for 6.8 ± 3.1 years). There is no significant difference in age (*p* = 0.774), height (*p* = 0.054), and years of training (*p* = 0.178) between the groups. This study was approved by the local Ethics Committee of Tsinghua University and written informed consent was obtained from all subjects before the scanning.

### Brain Imaging

All subjects were scanned in a 3.0 Tesla Philips Achieva scanner with a standard 32-channel head coil in the Center for Biomedical Imaging Research of Tsinghua University. During each scan sequence, subjects were told to relax but stay awake with their eyes closed. Headphones and foam were used to reduce the noise of the machine in order to protect the hearing of the subjects. Eight minutes of Echo Planar Imaging (EPI) sequence was used to acquire Blood oxygen level-dependent (BOLD) data and the parameters were as follows: TE = 30 ms; TR = 2,000 ms; flip angle = 90°; slices = 37 sagittal slices; slice thickness = 3 mm; voxel size = 2.87 mm^3^ × 2.87 mm^3^ × 3.50 mm^3^; image matrix = 80 × 80; FOV = 230 mm × 230 mm, slices were obtained starting from the bottom of the cerebellum to the top of the head. Structural T1-weighted images were also acquired with the parameters of: flip angle = 8°; slices = 180 sagittal slices; voxel size = 1 mm^3^ × 1 mm^3^ × 1 mm^3^; FOV = 230 mm × 230 mm, slices were obtained starting from the right side to the left side of the brain.

### Data Processing

Magnetic resonance imaging data were processed using the RESTplus 1.22 package^1^ based on MATLAB. Preprocessing was carried out in regular order and with default parameters. To exclude the influence of the instability of the initial MRI signal, the first five time points were removed. Slice timing and head motion correction were then carried out to correct the influence of within-scan acquisition time difference and the movement of the subject’s head. Subjects with a head motion of translation > 2 mm in any plane or rotation > 2 in any direction were excluded. Realigned images were then spatially normalized to the high-resolution T1-weighted images in the standard Montreal Neurological Institute (MNI) space and were resampled to a voxel size of 3 mm^3^ × 3 mm^3^ × 3 mm^3^ for the sake of group comparison. After that, spatial smoothing was then conducted with a Gaussian kernel of 6 mm^3^ × 6 mm^3^ × 6 mm^3^ of full width at half maximum (FWHM).

Voxel-based morphometry analysis was performed using VBM8 toolbox.^2^ First, the high-resolution images were normalized to standard space using the DARTEL method. Then, the normalized images were segmented into different tissues, namely, gray matter, white matter, and cerebrospinal fluid (CSF). In the end, the gray matter images were normalized to the MNI template and then smoothed with a Gaussian kernel of 4 mm^3^ × 4 mm^3^ × 4 mm^3^ of FWHM.

In this study, brain spontaneous activity patterns were evaluated by ALFF and DC. ALFF was calculated using RESTplus 1.22. In order to standardize the ALFF, we calculated the ratio of the power spectrum of the low-frequency (0.01–0.08 Hz) range to that of the entire frequency range, namely fALFF, which was suggested to reflect the intensity of regional spontaneous brain activity (Zou et al., 2008). A binary matrix including undirected edges was obtained by thresholding each correlation at a level of *r* = 0.25 and DC was then calculated based on these matrices. In the end, z-transformation was used to DC maps for the sake of statistical analysis.

### Group Comparison

Statistical analyses were conducted with the general linear model (GLM) implemented in the spm12 package.^3^ A two-sample *t*-test was used to determine whether there were significant differences in brain structural and functional plasticity characteristics between elite endurance and anaerobic group, namely, gray matter volume, fALFF, and DC. During the statistical analysis, gender, age, and training years were calculated as covariates. Results were reported when a voxel was significant at a level of *p* < 0.01. The multiple comparison error was corrected using Bonferroni correction implemented in the spm12 program, yielding a family-wise error rate (FWER) at *p* < 0.01.

## Results

**FIGURE 1:**
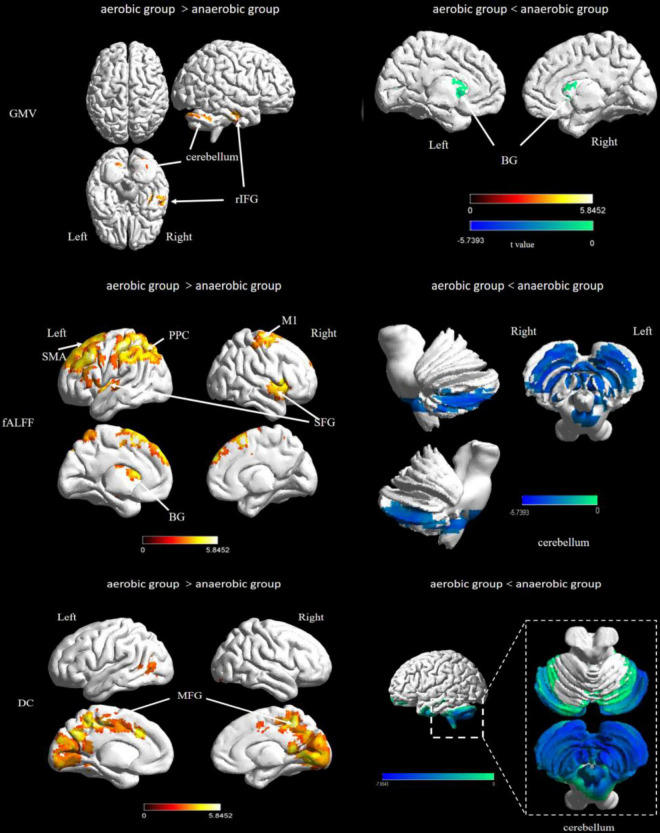
Difference of brain structural and functional plasticity characteristics between elite endurance and sprint athletes. ITG, inferior temporal gyrus; BG, basal ganglia; SFG, superior frontal gyrus; PFC, prefrontal lobe cortex; PPC, posterior parietal cortex; MFG, Middle Frontal Gyrus; OTC, occipitotemporal cortex.

The aerobic group showed higher gray matter volume in the region of the cerebellum and temporal lobe, including the right fusiform gyrus, inferior temporal gyrus, and bilateral cerebellum posterior lobe. In contrast, the anaerobic group demonstrated higher gray matter volume in the region of the basal ganglia, including the right caudate, the left claustrum, striatum, and thalamus (FWE corrected, *p* < 0.01, see Table 1 and Figure 1).

**TABLE 1:** Results of the voxel-based morphometry analysis.

Region	Side	BA area	Cluster *p* (FWE)	Cluster size	Peak coordinates			
					
					x	y	z	Peak *T*
Inferior temporal gyrus	R	20	<0.001	229	41	−18	−32	4.434
Inferior temporal gyrus	R	20	<0.001	240	57	−15	−35	4.345
Cerebellum posterior lobe	R	–	<0.001	501	9	−65	−47	4.194
Cerebellum posterior lobe	R	–	<0.001	1,108	38	−86	−26	5.020
Cerebellum posterior lobe	L	–	<0.001	350	−5	−74	−39	4.018
Cerebellum posterior lobe	L	–	<0.001	138	−20	−75	−33	4.605
Caudate	R	–	<0.001	168	30	−41	11	−5.739
Claustrum	L	–	<0.001	739	−33	−12	3	−4.885
Caudate, striatum, and thalamus	L	–	<0.001	552	−9	9	14	−4.505
Caudate	R	–	<0.001	735	17	11	11	−4.388

*Statistical maps were assessed at an uncorrected threshold of p < 0.01; the two clusters were significant at p < 0.01, familywise-error-corrected, at the cluster level. Coordinates are given in Montreal Neurological Institute (MNI) space. BA, Brodmann area; FEW, family-wise error. Peak T > 0 means aerobic group > anaerobic group, while peak T < 0 means aerobic group < anaerobic group.*

For the fALFF analysis, the aerobic group showed higher fALFF than the anaerobic group in the prefrontal cortex, the posterior parietal cortex and the basal ganglia (thalamus). In contrast, the anaerobic group demonstrated higher fALFF in the posterior cerebellum lobe (FWE corrected, *p* < 0.01, see Table 2 and Figure 1).

**TABLE 2:** Results of the amplitude of fractional low-frequency fluctuation analysis.

Region	Side	BA area	Cluster *p* (FWE)	Cluster size	Peak coordinates			
					
					x	y	z	Peak *T*
Prefrontal cortex	R	BA6	<0.001	6,403	30	−12	60	6.276
Posterior parietal cortex	L	BA5	<0.001	288	−9	−27	51	4.554
Thalamus	R	–	0.002	199	18	−36	6	3.936
Cerebellum posterior lobe	L	–	<0.001	6,788	−36	−84	−45	−7.000

*Statistical maps were assessed at an uncorrected threshold of p < 0.01; the two clusters were significant at p < 0.01, familywise-error-corrected, at the cluster level. Coordinates are given in MNI space. BA, Brodmann area; FEW, family-wise error. Peak T > 0 means aerobic group > anaerobic group, while peak T < 0 means aerobic group < anaerobic group.*

Degree centrality was significantly higher in the aerobic group than the anaerobic group in the frontal cortex and the medial part of the occipital and temporal lobe. In contrast, the anaerobic group demonstrated higher DC in the posterior cerebellum lobe (FWE corrected, *p* < 0.01, see Table 3 and Figure 1).

**TABLE 3:** Results of the degree centrality analysis.

Region	Side	BA area	Cluster *p* (FWE)	Cluster size	Peak coordinates			
					
					x	y	z	Peak *T*
Occipitotemporal cortex	R	BA19	<0.001	13,988	33	−60	0	5.029
Medial frontal gyrus	R	BA44	<0.001	3,232	51	9	15	5.084
Cerebellum posterior lobe	L	–	<0.001	24,941	−9	−60	−51	−7.315

*Statistical maps were assessed at an uncorrected threshold of p < 0.01; the two clusters were significant at p < 0.01, familywise-error-corrected, at the cluster level. Coordinates are given in MNI space. BA, Brodmann area; FEW, family-wise error. Peak T > 0 means aerobic group > anaerobic group, while peak T < 0 means aerobic group < anaerobic group.*

In order to show the strictness of the threshold used in this study, we extracted the GMV, fALFF, and DC signals at the peak point and then performed a *t*-test between two groups. The results will be reported in Supplementary Material. Effect-size *r* is stronger (*r* > 0.6) in the prefrontal cortex and cerebellum posterior lobe in fALFF analysis and the occipitotemporal cortex, medial frontal gyrus, and cerebellum posterior lobe in DC analysis.

## Discussion

Little research has directly reported on brain structural and functional plasticity related to different intensities of long-term exercise training. In the present study, we first compared the brain structural and functional plasticity characteristics between elite endurance and anaerobic group, i.e., two types of athletes who have participated in different intensity sports for a relatively long time. Results showed that the aerobic group demonstrated higher gray matter volume in the region of the cerebellum and temporal lobe, while the anaerobic group demonstrated higher gray matter volume in several areas of basal ganglia. The aerobic group also showed significantly higher fALFF and DC in the motor area of the frontal lobe and parietal lobe, and the frontal gyrus, respectively. At the same time, the anaerobic group demonstrated higher fALFF and DC in the posterior cerebellum lobe (FWE corrected, *p* < 0.01). The present results basically prove our hypothesis. However, several details are still worth further discussion.

### Aerobic Capacity, Endurance, and Brain Plasticity

Our results show that the aerobic group showed higher GMV in the temporal lobe and higher fALFF in the occipitotemporal than the anaerobic group. The aerobic group also showed stronger brain activity in the extensive prefrontal and medial frontal regions than the anaerobic group. This suggests that long-term aerobic and anaerobic exercise training may have different effects on the plasticity of the temporal lobe and frontal lobe. Individuals participating in aerobic exercise are often observed changes in the plasticity of the medial temporal lobe and the hippocampus (Van Praag et al., 1999; Erickson et al., 2011; Schlaffke et al., 2014). For example, Erickson et al. (2014) conducted a cross-sectional study and suggested that higher cardiorespiratory fitness is associated with higher GMV in the prefrontal cortex and hippocampus in orders. In addition, vast evidence has proved that engaging in aerobic exercise may cause beneficial changes in brain structure and function, whether in the elderly, young adults, or children. Raichlen et al. (2016) suggested that endurance running could enhance functional connectivity between the fronto-parietal network and frontal cortex and aerobic exercise could stress executive cognitive functions. Schaeffer et al. (2014) reported that 8-month instructor-led aerobic activities (e.g., tag or jump rope) could improve frontotemporal white matter integrity in overweight children. These studies compared the differences between people who have been engaged in endurance sports and a control group. In other words, it is not clear whether the plasticity in temporal lobe plasticity is related to aerobic exercise or just exercise. The current study directly compared aerobic and anaerobic athletes and proved that differences exist in GMV and fALFF in the temporal lobe and frontal lobe.

Aerobic athletes have relatively strong aerobic endurance (cardiorespiratory endurance or physical fitness) after years of endurance training. Previous studies have revealed the relationship between aerobic endurance and cerebellar gray matter volume in non-athletes. Aerobic athletes have higher cardiovascular requirements than anaerobic athletes. Anazodo et al. (2013) investigated the characteristics of brain gray matter volume in coronary artery disease patients with impaired aerobic capacity. Compared to healthy controls, coronary artery disease showed significant decreases GMV in the posterior cerebellum and cardiovascular rehabilitation was associated with the recovery of regional GMV in the posterior cerebellum (Anazodo et al., 2013). Recently, d’Arbeloff et al. (2020) investigated the associations between cardiovascular fitness and structural brain integrity in a cohort of 807 members. Results showed that aerobic endurance (indexed by VO2Max) is associated with greater cerebellar gray matter volume. This article suggests that cerebellar cortical plasticity may be associated with cardiovascular health-related neuroprotection.

Activity in the parietal lobe is often associated with movement observation (Buccino et al., 2001) and motor performance in elite athletes (Yarrow et al., 2009). Furthermore, in a recent study, the left parietal lobe was found to be associated with volitional qualities (Wei et al., 2020). Aerobic athletes may have greater willpower than anaerobic athletes because they need to perform relatively longer and harder exercises. Therefore, the aerobic group exhibited stronger ALFF in the parietal lobe.

### Anaerobic Capacity, Sprinting, and Brain Plasticity

According to Wenzel et al.’s (2014), rapid foot movement was accompanied by increased activation in the anterior cerebellum compared to slow foot movement. This result suggests that cerebellum activation increases when high-speed exercise is required. Our results showed that the anaerobic group demonstrated higher fALFF and DC in the cerebellum posterior lobe, which suggested that long-term anaerobic exercise might induce more plasticity changes in the cerebellum than aerobic exercise, allowing it to maintain strong spontaneous activities and functional connections. It is worth noting that we also found anaerobic group demonstrated lower GMV in the cerebellum posterior lobe. A possible explanation is the neural efficiency hypothesis (NEP). Experts have more effective cortical functions during the completion of the task, and their cortical activation shows the characteristics of spatial concentration (Del Percio et al., 2008). The saving of central resource consumption can reflect the high neurological efficiency of experts (Del Percio et al., 2011). In this current research, anaerobic athletes have enhanced the neural efficiency of related areas in the cerebellum during years of training, and need less gray matter volume to perform the tasks required during exercise. In other words, although the cerebellum of the anaerobic group is morphologically small, it showed a higher degree of activity and functional connectivity. In summary, our research further suggests the relationship between rapid whole-body movement and the cerebellum.

Additionally, our research suggests that long-term anaerobic exercise is probably related to the higher basal ganglia (especially in the caudate) GMV. Specifically, the aerobic group demonstrated lower gray matter volume in the region of the basal ganglia (the right caudate, the left claustrum, striatum, and thalamus) and higher fALFF than the anaerobic group in the basal ganglia (thalamus). As we know, basal ganglia are closely related to motor skill control. The basal ganglia are involved in monitoring the consequences of behavioral variations (Ross et al., 2003), movement programing and control, and interacting with some other motor-related cortex regions (Wu et al., 2004). Studies have also reported that patients with basal ganglia injury will have motor control problems, such as Parkinson’s disease (Dauer and Przedborski, 2003). Furthermore, Floyer-Lea and Matthews (2005) found that participating in long-term motor skill training could increase the activation of the motor cortical basal ganglia loop. Specifically, caudate is part of the corticobasal ganglia–thalamic loop and the reward system (Floyer-Lea and Matthews, 2005). Caudate is involved in spatial selection (Postle and D’esposito, 2003), tracking of behavioral states, and inhibitory control of motors (Arcizet and Krauzlis, 2018). Long-term participation in anaerobic motor skill training might lead to plasticity changes in the caudate nucleus.

One limitation of this study is the exact physiological significance of ALFF is not yet fully understood, so we need to be cautious when interpreting the current results.

## Conclusion

This study first compared the whole-brain structural and functional plasticity of aerobic and anaerobic athletes. The aerobic group showed higher GMV in the temporal gyrus and lower GMV in the basal ganglia. In addition, the aerobic group also showed significantly higher fALFF and DC in the motor area of the frontal lobe and parietal lobe, and the frontal gyrus, respectively. At the same time, the anaerobic group demonstrated higher fALFF and DC in the cerebellum posterior lobe. These results demonstrate that long-term participation in exercise of different intensities is related to different brain structural and functional plasticity characteristics. In addition, brain structural and functional plasticity characteristics are not completely consistent.

## Data Availability Statement

The raw data supporting the conclusions of this article will be made available by the authors, without undue reservation.

## Ethics Statement

The studies involving human participants were reviewed and approved by Ethics Committee of Tsinghua University. The patients/participants provided their written informed consent to participate in this study.

## Author Contributions

CC and YL: experimental design. KZ, TZ, LZ, JL, and RL: data collection. KZ: writing—original draft preparation. Y-KJ and KZ: writing—review and editing. CC: funding acquisition. All the authors discussed the results and agreed to the published version of the manuscript.

## Conflict of Interest

The authors declare that the research was conducted in the absence of any commercial or financial relationships that could be construed as a potential conflict of interest.

## Publisher’s Note

All claims expressed in this article are solely those of the authors and do not necessarily represent those of their affiliated organizations, or those of the publisher, the editors and the reviewers. Any product that may be evaluated in this article, or claim that may be made by its manufacturer, is not guaranteed or endorsed by the publisher.

## Footnotes

1
http://www.restfmri.net

2
http://dbm.neuro.uni-jena.de/vbm

3
https://www.fil.ion.ucl.ac.uk/spm/software/download/
