# Supplementary material for: Exercise Intensity and Brain Plasticity: What’s the Difference of Brain Structural and Functional Plasticity Characteristics Between Elite Aerobic and Anaerobic Athletes?
Source: Front Hum Neurosci. 2022 Feb 22;16:757522. doi: 10.3389/fnhum.2022.757522 (PMC8901604; doi:10.3389/fnhum.2022.757522)
Supplement: Supplementary file 1 [file Data_Sheet_1.docx]

TABLE 1. The effect size estimates of the GMV results

| Region | Peak coordinates | | | Aerobic Group (n = 23) | Anaerobic Group (n = 25) | *p*-value | Cohen's d | r |
| --- | --- | --- | --- | --- | --- | --- | --- | --- |
|  | x | y | z |  |  |  |  |  |
| Inferior Temporal Gyrus | 41 | -18 | -32 | 0.713±0.128 | 0.595±0.106 | 0.001 | 1.006 | 0.449 |
| Inferior Temporal Gyrus | 57 | -15 | -35 | 0.601±0.118 | 0.492±0.1 | 0.001 | 1.002 | 0.448 |
| Cerebellum Posterior Lobe | 9 | -65 | -47 | 0.835±0.118 | 0.745±0.108 | 0.009 | 0.793 | 0.369 |
| Cerebellum Posterior Lobe | 38 | -86 | -26 | 0.155±0.044 | 0.112±0.033 | <0.001 | 1.115 | 0.487 |
| Cerebellum Posterior Lobe | -5 | -74 | -39 | 0.781±0.109 | 0.713±0.124 | 0.051 | 0.580 | 0.278 |
| Cerebellum Posterior Lobe | -20 | -75 | -33 | 0.863±0.118 | 0.805±0.082 | 0.050 | 0.577 | 0.277 |
| Caudate | 30 | -41 | 11 | 0.088±0.016 | 0.102±0.016 | 0.003 | -0.908 | -0.413 |
| Claustrum | -33 | -12 | 3 | 0.74±0.065 | 0.788±0.072 | 0.019 | -0.702 | -0.331 |
| Caudate, striatum, thalamus | -9 | 9 | 14 | 0.31±0.055 | 0.364±0.039 | <0.001 | -1.114 | -0.487 |
| Caudate | 17 | 11 | 11 | 0.771±0.102 | 0.843±0.073 | 0.008 | -0.801 | -0.372 |
| TABLE 2. The effect size estimates of the fALFF results | | | | | | | | |
| Region | Peak coordinates | | | Aerobic Group (n = 23) | Anaerobic Group (n = 25) | *p*-value | Cohen's d | effect-size r |
|  | x | y | z |  |  |  |  |  |
| Prefrontal Cortex | 30 | -12 | 60 | 0.376±0.197 | -0.225±0.548 | <0.001 | 1.460 | 0.590 |
| Posterior Parietal Cortex | -9 | -27 | 51 | 0.32±0.295 | -0.131±0.517 | 0.001 | 1.069 | 0.471 |
| Thalamus | 18 | -36 | 6 | 0.397±0.213 | 0.124±0.392 | 0.005 | 0.867 | 0.398 |
| Cerebellum Posterior Lobe | -36 | -84 | -45 | -1.783±0.84 | -0.278±0.689 | <0.001 | -1.959 | -0.700 |
| TABLE3. The effect size estimates of the DC results | | | | | | | | |
| Region | Peak coordinates | | | Aerobic Group (n = 23) | Anaerobic Group (n = 25) | *p*-value | Cohen's d | effect-size r |
|  | x | y | z |  |  |  |  |  |
| Occipitotemporal Cortex | 33 | -60 | 0 | 0.833±0.582 | 0.003±0.501 | <0.001 | 1.530 | 0.608 |
| Medial Frontal Gyrus | 51 | 9 | 15 | 0.714±0.449 | -0.023±0.501 | <0.001 | 1.549 | 0.612 |
| Cerebellum Posterior Lobe | -9 | -60 | -51 | -1.582±0.441 | -0.183±0.807 | <0.001 | -2.152 | -0.733 |
